# Accuracy of the QIAxcel Automated System for MIRU-VNTR Genotyping of *Mycobacterium tuberculosis* in Two Limited Resource Settings

**DOI:** 10.3390/jcm9020389

**Published:** 2020-02-01

**Authors:** Silva Tafaj, Asma Ghariani, Alberto Trovato, Perlat Kapisyzi, Leila Essalah, Emna Mehiri, Gentian Kasmi, Genc Burazeri, Leila Slim Saidi, Daniela Maria Cirillo

**Affiliations:** 1 Microbiology Department, University Hospital “Shefqet Ndroqi”, 1044 Tirana, Albania; 2 National Reference Laboratory of Mycobacteria, A.Mami Hospital of Pulmonology, 2080 Ariana, Tunisia; ghariani.as@gmail.com (A.G.); essalahleila@hotmail.com (L.E.); emna.mehiri@tunet.tn (E.M.); leilaslimsaidi@gmail.com (L.S.S.); 3 Emerging Bacterial Pathogens Unit, Division of Immunology, Transplantation and Infectious Diseases, IRCCS San Raffaele Scientific Institute, 20132 Milan, Italy; trovato.alberto@hsr.it (A.T.); cirillo.daniela@hsr.it (D.M.C.); 4 Service of Clinical Microbiology, University Hospital “Mother Theresa”, 1005 Tirana, Albania; g.kasmi@hotmail.com; 5 Institute of Public Health, 1001 Tirana, Albania

**Keywords:** tuberculosis, MIRU-VNTR typing, molecular surveillance

## Abstract

Mycobacterial interspersed repetitive units variable number tandem repeat (MIRU-VNTR) typing of *Mycobacterium tuberculosis* complex (MTBC) isolates, based on 24 loci, is still widely used as the standard for routine molecular surveillance of tuberculosis (TB). QIAxcel system is proposed as an affordable tool that could replace conventional gel electrophoresis and provide high concordance with the reference methods regarding MIRU-VNTR typing of MTBC. We aimed to evaluate the QIAxcel accuracy for allele calling of MIRU-VNTR loci in two regional reference laboratories. A total of 173 DNA were used for the study. Results obtained with QIAxcel were compared to the reference results obtained with an ABI 3730 DNA analyzer. In Albania, the overall agreement with the reference method was 97.92%. A complete agreement result was obtained for 17 loci. In Tunisia, the overall agreement with the reference method was 98.95%. A complete agreement result was obtained for 17 loci. Overall agreement in both centers was 98.43%. In our opinion, use of QIAxcel technology has the potential to be reliable, given an optimized algorithm. Inaccuracies in sizing of long fragments should be solved, especially regarding locus 4052.

## 1. Introduction

Tuberculosis (TB) currently remains one of the top 10 global causes of mortality, as well as the leading cause of death from a single infectious pathogen [1]. In the host-pathogen interaction, genetic background of *Mycobacterium tuberculosis* complex (MTBC) does matter [2], alongside with other host and environmental factors. Some MTBC lineages are more successful than others at spreading and are more likely to be associated with resistance due to higher rates of acquiring resistance mutations [3]. Some genotypes are more likely to be transmitted and some are more virulent than others [4]. Thus, genotyping contributes substantially to TB epidemiology. Lineage classification, cluster investigation, and, at the same time, studies of phylogeny and evolution can be performed. This information combined with clinical and epidemiological data is very useful to understand dynamics of transmission in specific settings and should be the main driver for achieving TB control in European countries and worldwide.

Mycobacterial Interspersed Repetitive Units Variable Number Tandem Repeat (MIRU-VNTR) typing by the 24-loci is a mini-satellite typing system that was first proposed by Supply et al. (2006) as a new standard for first-line routine epidemiological discrimination of MTBC isolates [5] and it later replaced the former gold-standard IS6110 DNA fingerprinting by the restriction fragment length polymorphism (RFLP) technique. It is an important method to analyze the genetic diversity of clinical isolates. It is well standardized, relatively quick, and suitable for country surveillance purposes. VNTR products, provided by end-point PCR reactions, are analyzed using conventional gel electrophoresis or automated capillary electrophoresis. Data analysis and comparison of patterns is done either manually or using a special software. Conventional gel electrophoresis is cheap but somewhat cumbersome, slow, and can manifest problems related to inter- and intra-laboratory reproducibility [6,7], while automated capillary electrophoresis by sequencers can be quite an expensive tool.

The QIAxcel 12-channel capillary electrophoresis instrument utilizes disposable, multiple-use cartridges and provides an automated analysis of the results. Regarding MIRU-VNTR typing of MTBC isolates, the QIAxcel system is proposed as an affordable tool that could replace conventional gel electrophoresis and provide high concordance with the reference methods. A fairly recent multicenter evaluation study carried out in two large reference centers in UK and Italy [8] revealed that the QIAxcel system could be considered as an effective alternative in smaller reference and regional laboratories, offering good performance. In this context, we aimed to assess the QIAxcel accuracy for allele calling of MIRU-VNTR loci in two regional reference laboratories with limited resources and where agarose gel electrophoresis is used, but with reproducibility issues and low throughput.

Albania is a Southeastern European country with an upper-middle income economy according to the World Bank list of economies [9]. As of 1 January 2018, the Albanian population included about 2.87 million inhabitants [10]. In 2017 in Albania, a total of 503 TB cases were reported (WHO estimated rate of 20 [95% CI = 17–23] per 100,000 inhabitants); 69% pulmonary new cases and 61% laboratory confirmed cases of pulmonary. Estimated rate of multidrug-/rifampicin-resistant TB (MDR/RR-TB) cases was 2.3% (95% CI = 0.64%–5.8%) among new cases and 6.7% (95% CI = 0.17%–32%) among previously treated cases [9]. National Reference TB Laboratory (TB NRL) processes an average of 4000 samples per year for TB diagnosis.

Tunisia is a North African country with a lower-middle income economy. As of 1 July 2016, Tunisian population included about 11.3 million inhabitants [11]. In 2017 in Tunisia, a total of 3145 TB cases were reported (WHO estimated rate of 34 [95% CI = 26–43] per 100,000 inhabitants), 38% pulmonary new cases and 80% laboratory confirmed cases of pulmonary. Estimated rate of MDR/RR-TB cases was 1.1% (95% CI = 0.53%–2%) among new cases and 13% (95% CI = 6.1%–23%) among previously treated cases [12]. National Reference Laboratory of Mycobacteria (NRLM) processes an average of 19,000 samples per year for TB diagnosis.

## 2. Methods

### 2.1. Study Setting in Albania

The study was carried out simultaneously in Tirana, the capital of Albania and Ariana, Tunisia in 2016. In Albania, the study was conducted at the TB NRL in Tirana at the University Hospital “Shefqet Ndroqi,” which constitutes the largest national TB hospital and reference center of the country. A convenience sample of 90 native DNA belonging to the NRLs 2011 collection of MTBC was chosen for the study. DNA was manually extracted by thermal lysis of TE-suspended pellets of liquid MTBC cultures on a TE (TRIS-HCl 10 mM, EDTA 1 mM) buffer and stored at −20 °C. Before typing, DNA was purified and concentrated by a cold ethanol precipitation procedure.

### 2.2. Study Setting in Tunisia

In Tunisia, the study was conducted at the NRLM of the A. Mami Hospital of Pulmonology in Ariana. DNA belonged to 83 MTBC strains isolated from confirmed pulmonary TB in 2015. DNA was extracted and purified using the QIAmp DNA Blood mini kit (QIAGEN, Hilden, Germany).

### 2.3. QIAxcel Procedure

In both countries, PCRs were performed according to an optimized PCR protocol using primers as described by Supply et al. (2006) for a 24-loci panel. PCR products were uploaded in the QIAxcel instrument for separation of fragments and allele designation by electrophoresis under controlled conditions as per manufacturer’s instructions. QIAxcel DNA High Resolution Kit (1200) containing the QIAxcel DNA High Resolution Gel Cartridge was used for the study in both countries. A positive control (H37Rv) was included in each PCR and QIAxcel run. QX Alignment Marker (15 bp–5 kb) and QX DNA Size Marker (100 bp–2.5 kb) were included in every QIAxcel run. Allele calling was performed by using QIAxcel ScreenGel software. Results were entered into an Excel spreadsheet and analyzed further for accuracy and concordance of results.

### 2.4. Reference Method

All 173 DNA samples from both countries were analyzed in Milan, Italy by an automated sequencer, with a 3730 DNA analyzer (Applied Biosystems, Foster City, California, USA), as a reference method. Multiplex PCRs were performed using the 24 MIRU-VNTR typing kit (Genoscreen, Lille, France). Analysis of results was performed using the GenneMapper v3.7 software.

### 2.5. Assessment of the Accuracy of the QIAxcel Method

The accuracy of the QIAxcel method was assessed through the comparison with the reference method and the analysis of the overall concordance. Additionally, the standard deviation and the size deviation from the reference size were calculated. Wilcoxon signed-rank test, a non-parametric test, was used to compare mean ranks (paired differences) between each pair of alleles for Albania and Tunisia, separately. *p*-values ≤ 0.05 were considered as statistically significant. Statistical Package for Social Sciences (SPSS, version 19.0) was used for data analysis.

### 2.6. Ethical Considerations

No ethical permission for the study was sought as the MTBC DNA samples were anonymized and no patient information could be retrieved from them.

## 3. Results

In Albania, the mean age of patients was 44 (range 2–83) years old and the male to female ratio was 2:6 (65 to 25). In Tunisia, the mean age of patients was 40 (range 15–80) years old and the male to female ratio was 4:9 (69 to 14).

**Figure 1:**
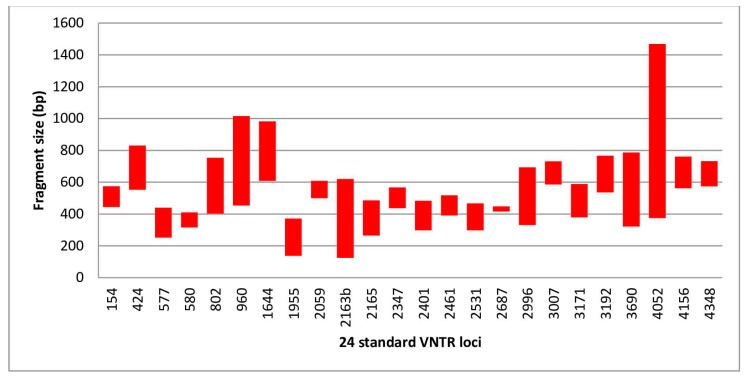
Sizes of PCR fragments determined by the QIAxcel system in all isolates.

A total of 173 DNA (90 in Albania and 83 in Tunisia) were compared to the reference method with a total of 4152 run assays. A PCR failure was observed in 14 assays (4 in Albania and 10 in Tunisia). The allele distribution and the molecular size ranges are summarized in the Table 1 and Figure 1. The molecular sizes ranged from 125 to 1469 bp and from 134 to 1229 bp in Albania and Tunisia, respectively. Smallest and largest sizes were observed in loci VNTR 2163b and VNTR 4052 at both centers.

In Albania, the overall agreement with the reference method was 97.92%. Forty-five discrepant results were obtained, of which 25 (55.6%) were in locus 4052. All discrepant results in locus 4052 had a molecular size equal or greater than 877 bp. Two discrepant results in locus 424, two in locus 960, five in locus 1644, two in locus 3690, and one in locus 4348 had a molecular size greater than 750 and the allele designation was one unit higher than the reference value. Only eight discordant results could not be explained with the large molecular weight, of which seven were in locus 960 (range 454–509 bp). The latter seemed to be a systematic error, the only one found in the study; seven discordant results with an underestimation of three units (QIAxcel allele designation 0/reference allele designation 3). We were not able to explain the reasons for this error. A complete agreement result was obtained for 17 loci. VNTRs 424, 960, 1644, 2687, 3690, 4052, and 4348 showed an agreement rate ranging from 72.22% to 98.89%.

In Tunisia, the overall agreement with the reference method was 98.95%. Twenty-one discrepant results were obtained. A complete agreement result was obtained for 17 loci. VNTRs 960, 2165, 300, 3192, 3690, 4052, and 4156 showed an agreement rate ranging from 91.57% for VNTR 4052 to 98.80% for VNTR 4156. Fourteen of the 21 discordant results (66,67%) had a molecular size higher than 700 bp. It was observed with VNTRs 960 (2/2), 3007 (1/2), 3192 (2/3), 3690 (2/4), 4052 (6/6), 4156 (1/1). Locus 4052 had the largest proportion of discordant result (27,27%) with a molecular size equal or greater than 742 bp in all cases. The miscalculation of the number of repeats by the QIAxcel was due to an under- or overestimation of the molecular size in 10 and five cases, respectively. A difference of one unit was observed in 11 discordant results. Seven discordances were due to a miscalculation of two to five units.

The analysis of all the discordant results is summarized in Table 2.

**Figure 2:**
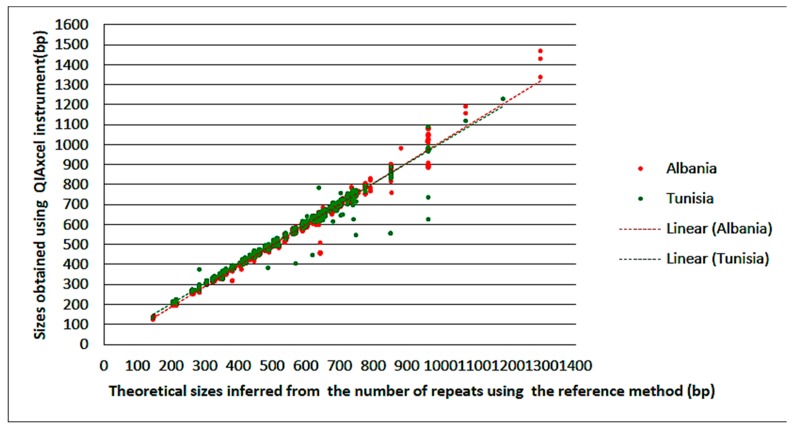
Sizing correlation between QIAxcel and reference ABI 3730 methods.

Figure 2 is a scatter plot representation of the theoretical sizes versus the sizes obtained using QIAxcel in both centers. Overall agreement in both centers was 98.43%. Agreement rates for each discordant locus and country are summarized in Table 3.

The observed ranking (order of values) of molecular weights obtained by QIAxcel in Albania was significantly different from their reference value for all loci, except for VNTR 802, 3007, 3690, 4052, 4156, and 4348. In Tunisia, the observed ranking of molecular weights obtained by QIAxcel was significantly different from their reference value for all loci.

## 4. Discussion

Currently, as next-generation sequencing (NGS) technologies of typing based on whole genome sequencing (WGS) seem to be the future of MTBC typing, many large reference centers are increasingly using all available genetic information these technologies offer. However, as we still miss standardization of WGS analysis pipelines, databases for sharing WGS data at a global level, and international agreement on the relevant genomic distances for cluster definition, these technologies will need more time to be implemented worldwide [13]. For the moment, these methods are more expensive than MIRU-VNTR typing and countries like Albania and Tunisia could benefit from using the QIAxcel technology.

In both sites, the QIAxcel system was easy to introduce. A two-day, on-site training was enough for staff to become familiar with the MIRU-VNTR genotyping of MTBC. It was also a lot faster to get the results compared to the MIRU-VNTR manual gel-electrophoresis procedure. Although cost calculation was not among the objectives of this study, cost information collected in Albania and Tunisia show that cost of QIAxcel typing of MTBC (including QIAxcel consumables, QIAxcel instrument depreciation, and PCR reagents) is less than cost of genotyping by automated sequencers.

As 37 out of 45 and 5 out 21 discrepancies observed in Albania and Tunisia were due to an overestimation of the molecular size and subsequently erroneous allele designation (one unit higher), algorithm optimization of QIAxcel technology would make this method very useful in these settings. It would increase accuracy to 99.63% in Albania and 99.19% in Tunisia with a very high overall rate of concordance of 99.41%. These results are in line with other studies performed so far on MIRU-VNTR typing of MTBC with the QIAxcel system [14,15].

The QIAxcel system is an accurate method that detects allele lengths with a precision of 5 bp in more than half of the cases. For a proper allele calling, the maximum sizing deviation must not exceed half of the shortest repeat length [15]. Sizing deviation did not exceed 20 bp in Tunisia. However, in Albania, size deviations of more than 20 bp could explain the discrepant result observed with VNTR 1644 allele 5 and VNTR 4052 alleles 7, 8, and 11. Locus 4052 also showed the highest standard deviation which may be related to its high molecular size.

The new approach to variable number tandem repeats (VNTR) analysis using the QIAxcel capillary electrophoresis system and a software-integrated peak calling function was first reported in Japan in 2013 [14]. The multicenter study conducted in two references centers in London and Milan [8] compared the QIAxcel genotyping results with the reference technique and demonstrated that PCR fragment sizes varied significantly depending on the number of copies within specific VNTR loci with the shortest and longest fragments. These variations particularly affected loci 2163b and 4052. According to the authors of this study, the accuracy depended primarily on the PCR fragment length being suboptimal for sizes >750 bp [8]. These problems could be both loci-specific and PCR fragment size-dependent, as observed in our study. As previously recommended, an optimization of separation procedures and peak calling algorithms, especially for the longest fragments, are necessary [8].

A PCR failure was observed in four assays in Albania and 10 in Tunisia. In Milan, PCR failure was confirmed for all four assays in Albania and only one out of ten in Tunisia. This may be related to different DNA extraction methods used for the study in Albania and Tunisia, hence an optimized protocol for DNA extraction could be of benefit to the users of QIAxcel methodology.

## 5. Conclusions

Seemingly, use of QIAxcel technology may be reliable and easy to use in regional reference TB laboratories, given a good DNA quality and optimized algorithm. Inaccuracies in sizing of long fragments should be solved, especially regarding locus 4052. MIRU-VNTR typing of MTBC, used in a timely manner, could effectively assist the control of susceptible and drug-resistant TB in Albania and Tunisia.

## Figures and Tables

**Table 1:** Allelic distribution of all isolates (nr = 173).

Locus	Size (bp)		Allele Distribution										
	Minimum	Maximum	0	1	2	3–3s	4	5	6	7	8	9	10
154	445	574		19	151	2							
424	553	830	1	4	119	19	24	6					
577	252	440			12	74	85	2					
580	316	410			141	32							
802	403	754		1	38	52	37	32	12	1			
960	454	1016		3	15	55	40	58		1		1	
1644	608	982		33	12	104	23		1				
1955	138	371		3	91	77		2					
2059	500	608		19	153								
2163b	125	620		6	41	75	23	11	16		1		
2165	265	486		3	73	91	6						
2347	437	567			53	2	118						
2401	298	483		18	89	11	55						
2461	392	518		3	164	6							
2531	298	467				51		109	13				
2687	416	448		173									
2996	331	693		4	2	7	13	115	18	3	11		
3007	586	731			25	144	4						
3171	380	589		5	17	147	3	1					
3192	536	767		4	79	72	16	2					
3690	322	787		3	18	99	12	17	11	2	6		
4052	375	1469			2	16	35	45	27	33	5		4
4156	563	761		44	109	19							
4348	574	732		1	169	2	1						

**Table 2:** Discrepancies between QIAxcel and ABI 3730 results in all isolates.

Locus	No. of Isolates	Site	QIAxcel		ABI 3730	
			Size (bp)	Inferred No. of Repeats	Reference Size(bp)	Inferred No. of Repeats
424	2	Albania	821, 830	6	792	5
960	2	Tunisia	714, 716	4	749	5
960	2	Albania	890, 1016	8, 10	855, 961	7, 9
960	7	Albania	454, 457, 458 (2), 459 (2), 509	0	643	3
1644	5	Albania	804, 805 (2), 806, 982	5, 5, 5, 5, 8	777, 883	4, 4, 4, 4, 6
2165	2	Tunisia	405, 406	3	570, X	5, X
2687	1	Albania	416	0	447	1
3007	2	Tunisia	641, 650	3	604, 710	2, 4
3192	3	Tunisia	622, 646, 747	2, 3, 5	651, 704, 704	3, 4, 4
3690	2	Albania	784, 787	9	736	8
3690	4	Tunisia	556, 556, 564, 447	5, 5, 5, 3	852, 852, X, 620	10, 10, X, 6
4052	5	Albania	877, 881, 894 (2), 903	7	853	6
4052	15	Albania	1010, 1022, 1025, 1026, 1036, 1045, 1046, 1048, 1051, 1053, 1077, 1079, 1081, 1082, 1089	8	964	7
4052	2	Albania	1157, 1191	9	1075	8
4052	2	Albania	1338, 1430	11	1297	10
4052	1	Albania	1469	12	1297	10
4052	2	Tunisia	626, 626	4	742, 964	5, 7
4052	1	Tunisia	736	5	964	7
4052	1	Tunisia	888	7	853	6
4052	2	Tunisia	1090, 986	8	964	7
4052	1	Tunisia	1229	10	1186	9
4156	1	Tunisia	697	2	740	3
4348	1	Albania	732	4	699 < > 752	3 < > 4

**Table 3:** Concordance for discrepant variable number tandem repeat (VNTR) loci in each center and overall.

Concordance (%)			
Locus	Albania	Tunisia	Overall
424	97.78	100	98.89
960	90.00	97.59	93.80
1644	94.44	100	97.22
2165	100	97.59	98.80
2687	98.89	100	99.44
3007	100	97.59	98.80
3192	100	96.39	98.19
3690	97.78	95.18	96.48
4052	72.22	91.57	81.89
4156	100	98.80	99.40
4348	98.89	100	99.44
